# Advances in Molecular Mechanisms of Wheat Allergenicity in Animal Models: A Comprehensive Review

**DOI:** 10.3390/molecules24061142

**Published:** 2019-03-22

**Authors:** Yining Jin, Harini G. Acharya, Devansh Acharya, Rick Jorgensen, Haoran Gao, James Secord, Perry K. W. Ng, Venugopal Gangur

**Affiliations:** 1 Department of Food Science and Human Nutrition, Food Allergy & Immunology Laboratory, Michigan State University, East Lansing, MI 48824, USA; yining@msu.edu (Y.J.); nannaemail@hotmail.com (H.G.A.); devanshacharya@gmail.com (D.A.); jorgen70@msu.edu (R.J.); gaohaora@msu.edu (H.G.); secordj1@msu.edu (J.S.); 2 Cereal Science Laboratory, Department of Food Science and Human Nutrition, Michigan State University, East Lansing, MI 48824, USA; ngp@msu.edu

**Keywords:** wheat allergenicity, wheat hypersensitivity, food allergy, food allergen, food chemistry, IgE, animal model, wheat anaphylaxis, molecular mechanisms, food safety

## Abstract

The prevalence of wheat allergy has reached significant levels in many countries. Therefore, wheat is a major global food safety and public health issue. Animal models serve as critical tools to advance the understanding of the mechanisms of wheat allergenicity to develop preventive and control methods. A comprehensive review on the molecular mechanisms of wheat allergenicity using animal models is unavailable at present. There were two major objectives of this study: To identify the lessons that animal models have taught us regarding the molecular mechanisms of wheat allergenicity and to identify the strengths, challenges, and future prospects of animal models in basic and applied wheat allergy research. Using the PubMed and Google Scholar databases, we retrieved and critically analyzed the relevant articles and excluded celiac disease and non-celiac gluten sensitivity. Our analysis shows that animal models can provide insight into the IgE epitope structure of wheat allergens, effects of detergents and other chemicals on wheat allergenicity, and the role of genetics, microbiome, and food processing in wheat allergy. Although animal models have inherent limitations, they are critical to advance knowledge on the molecular mechanisms of wheat allergenicity. They can also serve as highly useful pre-clinical testing tools to develop safer genetically modified wheat, hypoallergenic wheat products, novel pharmaceuticals, and vaccines.

## 1. Introduction

Food is essential for life. Consequently, the host immune system has evolved mechanisms to establish immune tolerance to food-derived protein antigens and allergens [1,2]. However, a growing number of humans are losing immune-tolerance to several foods, including wheat, as evidenced by an ongoing epidemic of food allergies (also known as hypersensitivity) [2,3]. According to the government food regulators in the USA, Canada, Europe, Australia, New Zealand, and Japan, there are 7 to 14 common foods, including wheat, that are known to trigger life-threatening food allergic reactions known as anaphylaxis [1,2,4,5,6,7].

**Figure 1:**
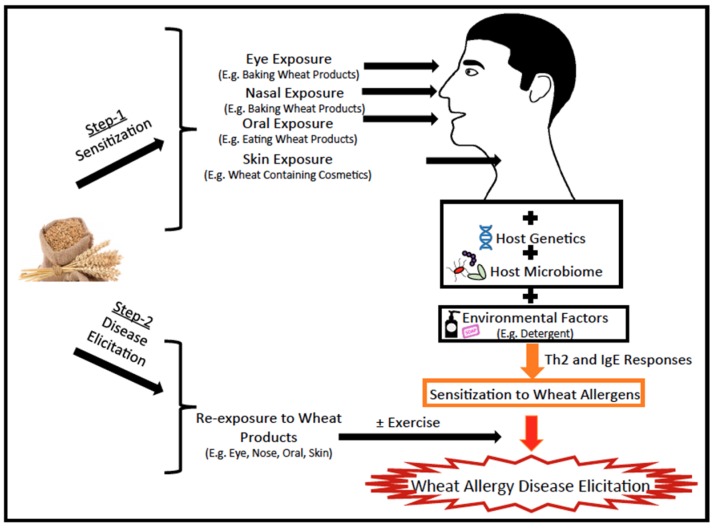
The genesis of wheat allergy: sensitization and elicitation of disease. Development of wheat allergy involves two steps: Step-1: Exposure of genetically susceptible subject to wheat products via the eye, nasal, oral, and skin routes in the context of a dysregulated host-microbiome and additional environmental co-factors, such as detergents in facial soap containing wheat allergens, activate the T helper (Th)-2 immune responses with consequent IgE antibody production. These antibodies load the mast cells and basophils, resulting in the immune state termed as sensitization. Step-2: Re-exposure of sensitized subjects to wheat results in the binding of allergens to IgE-loaded mast cells and basophils that release histamine and other inflammatory mediators, causing clinical symptoms of disease (diarrhea, vomiting, hives, rashes, dermatitis, conjunctivitis, rhinitis, asthma, or anaphylaxis). In some cases, exercise after exposure to wheat can trigger anaphylaxis in sensitized subjects.

Prevalence of wheat allergies has increased from 0.2% to 0.4% in the USA between 1996 and 2007 [8,9]. However, mechanisms underlying wheat allergies are not well understood at present. The genesis of wheat allergy is thought to be similar to other food allergies involving two steps: Sensitization to wheat allergens and wheat allergy disease elicitation among sensitized subjects (Figure 1) [1,2,3]. Sensitization of genetically susceptible hosts occur upon exposure to wheat products (via the eye, nasal, oral, skin routes) in the context of a dysregulated host microbiome and environmental co-factors (e.g., detergents in wheat containing cosmetics, such as facial soap) results in activation of T helper (Th)-2 lymphocyte responses with consequent IgE antibody production [1,2,3]. Re-exposure of sensitized subjects to wheat results in elicitation of disease; in some cases, exercise after re-exposure can trigger the disease (Figure 1) [1,2,3].

**Figure 2:**
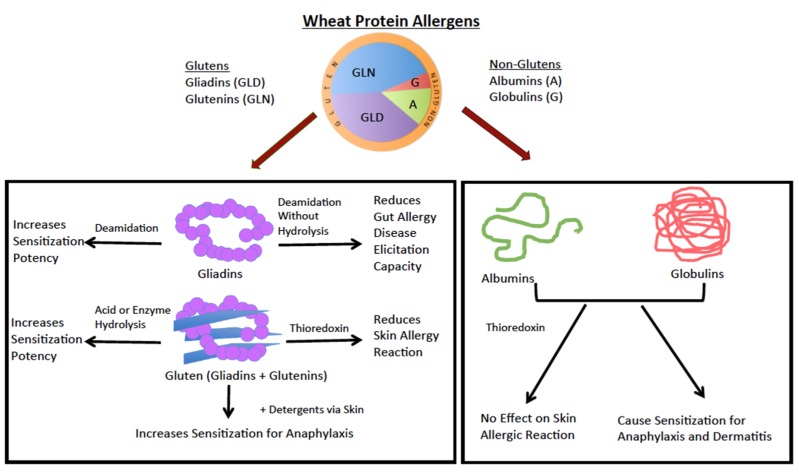
Wheat allergens: effect of molecular modifications on wheat allergenicity in animal models. Wheat contains 10%–18% protein by a dry weight basis. The top part of the figure shows the relative composition of four groups of wheat protein allergens. These include gluten—alcohol-soluble gliadins (or prolamins) (30%–40%) and acetic acid or alkali-soluble glutenins (or glutelins) (45%–50%), and non-gluten proteins, such as water-soluble albumins (10%–12%) and saline-soluble globulins (5%–8%). The bottom part of the figure shows the effect of molecular changes induced by chemical and enzyme treatment on wheat protein allergenicity in animal models.

Among the major allergenic foods, wheat is the most cultivated crop in the world [10,11]. Food allergens in general are water/saline soluble proteins. However, wheat contains four different classes of protein allergens: Water-soluble (albumins), saline-soluble (globulins), alcohol-soluble (gliadins), and acid-soluble (glutenins) protein allergens (Figure 2). Gliadins are the most abundant proteins followed by glutenins, albumins, and globulins. The term, gluten, includes both gliadin and glutenin (Figure 2).

Wheat allergies are of two types: IgE-dependent reactions and IgE-independent, but eosinophil-dependent reactions; however, most wheat allergies are IgE-dependent reactions [12,13,14,15]. The IgE-mediated wheat allergies include three groups of disorders: (i) Occupational allergies, such as allergic rhinitis (AR), allergic conjunctivitis (AC), bakers asthma (BA), and contact urticaria (CU); (ii) wheat food allergy (WFA), such as atopic dermatitis (AD), gastrointestinal allergic disease, and systemic anaphylaxis [12,13,14,15]; and (iii) wheat-dependent exercise-induced anaphylaxis (WDEIA) [16,17,18]. The IgE-independent, but eosinophil-mediated wheat allergies include eosinophilic esophagitis (EOE) and eosinophilic gastritis (EOG) [19,20,21]. Since there is no animal model for wheat-induced EOE/EOG at present, this study deals only with the IgE-mediated wheat allergy.

Wheat allergies are often confused with celiac disease and non-celiac gluten sensitivity. In contrast to wheat allergies that are mediated by IgE antibodies, celiac disease is an autoimmune disease of the intestine, skin, and the brain; the non-celiac gluten sensitivity is mediated by the over-active innate immune system [22]. There are excellent reviews on animal models of celiac disease and non-celiac gluten sensitivity disorders [23,24,25]. However, a comprehensive review on the molecular mechanisms of wheat allergenicity using animal models is unavailable at present. The absence of such an article is a major barrier to the advancement of basic and applied research in wheat allergenicity, and this serves as the rationale for conducting this study. 

There were two major objectives of this study: (i) To identify the lessons that animal models have taught us regarding the molecular mechanisms of wheat allergenicity; and (ii) to identify the strengths, challenges, and future prospects of animal models in basic and applied wheat allergy research. 

We conducted a comprehensive review of the literature without date limits using the PubMed and Google Scholar databases and the following key words in various combinations: Food, wheat, hypersensitivity, allergy, food allergy, asthma, anaphylaxis, and animal model. Only English language articles were included. Any article that focused on wheat allergy was included in this study. Those articles that focused on animal models of celiac disease, non-celiac gluten sensitivity, or other food allergies were excluded. The selected articles were assigned to different authors to compile data on the specific questions asked. These articles were critically evaluated to achieve the objectives of this study. The rest of the reference articles cited were used for discussion purposes.

We report that although animal models have inherent limitations, they are critical to advance knowledge on the molecular mechanisms of wheat allergenicity. They can also serve as highly useful pre-clinical testing tools to develop safer genetically modified wheat, hypoallergenic wheat products, novel pharmaceuticals, and vaccines.

## 2. Insights in to the Molecular Mechanisms of Wheat Allergenicity as Revealed by Animal Models

### 2.1. Animal Models of Wheat Allergy: How Many Are There and What Lessons Have They Taught us?

An animal model for wheat allergy was first developed using dog, and subsequently using mouse and rat species. Here, we systematically compared and contrasted these models and identified the specific lessons that they have taught us about the molecular mechanisms of wheat allergenicity.

#### 2.1.1. Lessons from the Dog Model of Wheat Allergenicity

Buchanan et al. (1997) used inbred high IgE responder dogs (spaniel/basenji) that had been genetically selected for over 15 years for showing allergy to pollens and foods [26]. They developed a complex protocol to study the molecular nature of wheat allergenicity (Table 1). A remarkable feature of this model is that wheat-sensitized dogs developed vomiting and/or diarrhea when fed with wheat. 

The two major lessons learnt from this model on the molecular nature of wheat allergenicity are: (i) All four types of wheat proteins, namely albumins, globulins, gliadins, and glutenins, can elicit IgE and cause skin reactions in the following order of potency: Gliadins > glutenins > albumin > globulin; among gliadins, α/β-gliadins were most potent; and (ii) thioredoxin (an intra-chain disulfide bond reducing agent) treatment can mitigate the allergenicity of gliadins (including α/β and γ types) and the glutenin; however, thioredoxin gave less consistent effects on the allergenicity of albumins and globulins. They proposed that the thioredoxin method might be used in the production of hypoallergenic wheat-based foods (Figure 2).

#### 2.1.2. Lessons Learnt from the Mouse Models of Wheat Allergenicity

Several mouse models of wheat allergy have been developed during 2006–2017 (Table 2). Below is a discussion of how these models have advanced our knowledge on the molecular nature of wheat allergenicity. 

Kozai et al. (2006) developed a mouse model to explain the molecular mechanisms of wheat-dependent exercise-induced anaphylaxis (WDEIA) (Table 2, Table 3 and Table 4) [27]. They sensitized B10. A mice with albumin/globulin, gliadin and glutenin fractions. Then, they tested the effect of exercise (treadmill) after oral feeding with each protein fraction. This model showed that: (i) Gliadins and glutenin not only elicited sensitization, but also caused WDEIA; (ii) salt-soluble proteins neither caused sensitization nor WDEIA; (iii) exercise caused mucosal lesions after oral challenge with wheat proteins and the leakage of gliadin and glutenin proteins into the liver. Thus, gluten proteins (gliadin and glutenin) were linked to WDEIA.

Although ω5-gliadin was linked to WDEIA (above study), whether it can cause anaphylaxis independent of exercise was unknown. Tanaka et al. (2011) developed a mouse model to address this question (Table 2, Table 3 and Table 4) [28]. The soft wheat four was used to extract gliadin fraction and then it was used to purify ω5-gliadin. They used female B10.A mice for sensitization with gliadin using alum as an adjuvant. Sensitization was confirmed by IgE response. Then, mice received oral gavage with total gliadin or ω5-gliadin and were checked for anaphylaxis without exercise. They found that: (i) When total gliadin was injected into mice, most of the IgE antibody elicited actually binds to ω5-gliadin; and (ii) mice developed identical anaphylaxis reactions when orally fed with either gliadin or ω5-gliadin. Thus, gliadins can cause anaphylaxis even without exercise in mice.

Bodinier et al. (2009) wanted to find out how the allergic response in mice is different from that in wheat allergic humans [30]. To address this, they compared the allergic response in mice vs. wheat allergic children and adults. They tested the allergenicity of gliadin extract (GE) in different mice strains (Balb/c, C3H/HeJ, and B10.A) (Table 2, Table 3 and Table 4). Allergenic (IgE) antibody responses against total gliadin and purified gliadins (α/β, γ, ω1, 2, ω5) were compared. They also studied airways and spleen allergic responses. 

They found that: (i) Balb/c mice exhibited the strongest allergic responses in the blood and spleen (IgE, IL-4/IL-5) and the airways’ allergic responses; the other two mice strains did not show disease because they had very little immune response to gliadin. This evidence showed that allergic responses in mice are genetically controlled; (ii) all five fractions of gliadins were allergenic in Balb/c similar to wheat allergic children; the order of allergenicity of gliadin fractions in Balb/c mice was similar to that noted in children as follows: α/β > γ > ω1,2 > ω5. This order also corresponds to the relative abundance of these fractions in total gliadin extract. Interestingly, among wheat allergic adults, as opposed to Balb/c mice, ω5 was the major allergen; and (iii) most IgE epitopes were against the conformational (discontinuous) epitopes because the alum adjuvant used in this study favored this type of response [31].

In a follow-up study, Denery-Papini et al. (2011) mapped the IgE epitopes on gliadin and LTP1 in Balb/c mice vs. humans (Table 3) [29]. Using the pepscan technique, IgE epitopes were mapped. They used reduced and alkylated forms of proteins to identify continuous IgE epitopes. They found only one continuous IgE epitope on LTP1 shared by mice and humans (Table 3). Other IgE epitopes on LTP1 were conformational (or discontinuous) in nature in both species. In contrast, on ω5-gliadin, they found many continuous (or linear) epitopes in both species. A similar trend was noted, but to a lesser extent, for other gliadins and the low molecular weight (LMW)-glutenin subunit. Thus, they concluded that the IgE epitopes on gliadins and LTP1 recognized by Balb/c mice are similar to those recognized by wheat allergic humans.

Deamidation of gluten is a common practice used by the food industry because this modification of gluten increases its solubility, thus making it a preferred form of gluten to use as a food ingredient and in cosmetics. However, there are concerns on the potentially enhanced allergenicity of such modified gluten. Studies show that DG sensitizes mice more effectively than the native gliadin and that the DG elicited IgE profile in mice was very similar to that seen in wheat allergic humans. Two mouse model studies evaluated this elegantly as discussed below. 

Gourbeyre et al. (2012) used the Balb/c mouse model to test this hypothesis (Table 2 and Table 4) [32]. They found that: i) Native gliadin elicited a higher T helper 1 type of immune response and the deamidated gliadin (DG) elicited a higher T helper 2 or allergic immune response and histamine response. However, both types of proteins elicited anaphylaxis to the same extent; ii) native gliadin elicited IgE against all five gliadins (α/β, ω1/2, and ω5). However, these antibodies did not bind to DG. In contrast, DG elicited IgE against all five deamidated gliadins, which also bound the native gliadin. These data support that DG is more allergenic than the native gliadin.

Abe et al. (2014) used a different method to study the oral allergy disease elicitation potency of DG (Table 2 and Table 4) [33]. They produced a novel type of DG using carboxylated cat ion-exchange resins that does not cause peptide bond hydrolysis or polymerization. They did not test its sensitization capacity. Instead they tested its ability to elicit disease in mice that had already been sensitized to the native gliadin. Interestingly they found that oral administration of DG to native gliadin sensitized mice results in less allergic disease compared to the native gliadin. In summary, the authors suggested that deamidation of gluten by cation exchange treatment is a promising method for the production of hypoallergenic bread and cakes with better expansibility (Figure 2).

Hydrolyzed wheat protein (HWP) is widely used in cosmetics, such as soaps (Figure 1). There are reports of allergic reactions to HWP in humans by oral and skin exposure [34,35,36,37]. Therefore, Adachi et al. (2012) tested whether HWP elicited allergenicity in Balb/c mice via skin exposure without adjuvant (Table 2, Table 3 and Table 4, Figure 2) [38]. They exposed mice via skin to native gluten vs. HWP vs. HWP plus detergent (0.5% sodium dodecyl sulfate) vs. native gluten plus detergent using a published protocol that had been used previously for other food allergens [39,40,41,42,43].

They found that: (i) The skin exposure to native gluten does not induce sensitization for anaphylaxis; however, in the presence of a detergent, native gluten can induce sensitization for anaphylaxis. This suggested that the use of wheat protein in cosmetics can enhance wheat allergenicity; (ii) HWP with or without detergent can cause sensitization for anaphylaxis. HWP alone was allergenic via skin because of enhanced permeability due to increased solubility of protein by hydrolysis (Table 2 and Table 4). Thus, skin exposure to gluten with detergent and to HWP alone or with detergents (such as in cosmetics) is a plausible route of human sensitization for wheat anaphylaxis (Figure 1 and Figure 2). 

The mouse model reviewed above mostly used alcohol-soluble gliadins in testing. As discussed in the introduction, salt-soluble wheat proteins (SSWPs) are also important allergens in wheat allergic humans [44]. Therefore, Jin et al. (2017) tested whether SSWPs can elicit allergic disease in Balb/c mice similar to humans (Table 2 and Table 4) [45]. They found that: (i) SSWP elicits robust IgE responses and sensitization, and mice show anaphylaxis when challenged; (ii) they showed for the first time that in this mouse model, anaphylaxis is linked to IgE-mediated mucosal mast cell degranulation [45,46]; and (iii) interestingly, some of the wheat allergic mice (but not all) developed atopic dermatitis lesions on the face, showing extensive mast cell degranulation and elevated levels of pro-inflammatory and allergic chemokines and cytokines (Table 2 and Table 4). 

In summary, these mouse models have significantly advanced our knowledge on molecular mechanisms of wheat allergenicity (Figure 2, Table 4).

#### 2.1.3. Lessons from the Rat Models of Sensitization to Wheat Allergens

There are two rat models reported in the literature studying the mechanisms of wheat gluten allergenic sensitization (Table 1). Kroghsbo et al. (2014) bred Brown Norway (BN) for three generations on a gluten-free diet producing ‘gluten-free’ rats [47]. They compared the immunogenicity (IgG) and allergenicity (IgE) of native gluten vs. acid hydrolyzed gluten (AHG) vs. enzyme hydrolyzed gluten (EHG) (Table 1 and Table 4, Figure 2). They found that: (i) EHG was more immunogenic followed by AHG and native gluten; and (ii) in the oral exposure study, EHG was more allergenic than the native gluten and AHG. Thus, they found that only enzyme hydrolysis enhances the allergic sensitization (IgE) capacity of gluten by the oral route (Table 1 and Table 4, Figure 2). They also found that acid hydrolysis of gluten results in the generation of novel IgG binding epitopes.

Using a skin sensitization method, Ballegaard et al. (2019) studied the allergenicity of native gluten vs. AHG in wheat-tolerant mice fed with wheat diet vs. naïve mice that had been on a wheat-free diet [48]. They found that in naïve rats that were not wheat-tolerant, both glutens elicited allergenic responses. However, in wheat-tolerant rats that had been fed a wheat diet, only AHG elicited an allergenic antibody response, but at much lower levels (Table 1 and Table 4, Figure 2). Using inhibition assays, they found that acid-hydrolysis causes new epitope formation and therefore these new epitopes can now elicit an antibody response even in mice that were tolerant to native gluten.

In summary, these two elegant rat model studies have advanced our knowledge on the mechanisms of allergenic sensitization to hydrolyzed wheat gluten at the molecular epitope level (Figure 2, Table 4).

### 2.2. Animal Models of Wheat Allergy: What Are the Current Challenges and Opportunities?

Compared to other food allergy animal model development (e.g., peanut allergy), animal models are underutilized at present to advance basic and applied research on the mechanisms of wheat allergy. To facilitate future research and development, we have discussed below major challenges facing this area of science. 

#### 2.2.1. Current Challenges Facing Wheat Allergy Animal Models

Although no single animal model is expected to completely simulate the complex human disease of wheat allergy, currently existing animal models provide opportunities to improve (Table 5). Here, we have discussed a number of experimental variables to take into consideration for further improvement of the wheat allergy animal models in research and development.

##### Species, Sex, Age, and Strain of the Animals Used in Model Development

As reviewed earlier, three animal species (dog, mouse, and rat) have been used so far for wheat allergy model development (Table 2, Table 3 and Table 4). They all have their own strengths and limitations (Table 5). It appears that whereas dogs can develop wheat allergy disease naturally, like humans, mice can be made to develop wheat allergy disease only under experimental conditions. So far, disease readouts (e.g., anaphylaxis, skin/gut/airways reactions) have not been developed in rats for wheat allergy. It is noteworthy that dogs are particularly suitable to study the vomiting response; mice/rats do not develop this phenotype [26].

Overall, mice are preferred in model development for several reasons, including cost and reagents’ availability (Table 5). Both B10.A and Balb/c mice strains have been used in wheat allergy model development. However, the latter are preferred by most. There are conflicting reports of whether B10.A does or does not develop an IgE response to gliadins [27,28]. In particular, Balb/c mice show immune responses that are remarkably similar to that of wheat allergic humans [30,45]. Interestingly, C3H/Hej mice, which are popular peanut allergy models, do not develop a gliadin allergy, suggesting the possibility of genetic resistance [30]. However, other wheat allergens have been tested so far.

Use of ‘gluten-free’ BN rats for wheat allergenic sensitization testing is very encouraging. They are a good choice for inducing oral sensitization without adjuvants. However, disease elicitation readouts (e.g., anaphylaxis, gut reactions, skin reactions, etc.) have not been developed yet (Table 5). It is also unknown how they respond to non-gliadin wheat allergens. 

Currently, it is unknown whether sex and age influences the development of wheat allergies in humans [2,44]. Only female mice have been used for wheat allergy development for reasons not clearly stated (Table 2, Table 3, Table 4 and Table 5). However, female rats develop stronger responses to wheat compared to male rats [47]. 

Due to technical limitations, the use of mice pups of less than 4 weeks for model development is a difficult task facing researchers in this area at present. It may be necessary to develop technology to study wheat allergy in mice younger than 4 weeks to simulate childhood wheat allergy.

##### Routes of Sensitization and Elicitation of Disease

In humans, it is generally thought that oral, nasal, and skin routes of exposure lead to wheat allergy. However, animal models can be used to study this critical issue. Currently, most animal models have used intraperitoneal (IP) injections in sensitization with the exception of one mouse and one rat model, where skin sensitization was used, and one rat model, where oral sensitization was studied [38,47,48] (Table 5). The mouse models used injections to elicit anaphylaxis. Therefore, it is important to improve these models by developing oral challenge protocols to induce disease because the underlying mechanisms of disease elicitation in injection vs. oral challenge might be different.

##### To Use or Not-to-Use Adjuvants for Wheat Allergenicity Testing?

With the exception of one mouse and one rat study, all other animal models of wheat allergies have used alum adjuvant for inducing sensitization (Table 2, Table 3, Table 4 and Table 5). Use of alum adjuvant can provide robust phenotypes. However, humans are not exposed to wheat allergens along with alum adjuvant. In that sense, adjuvant-free models, such as skin sensitization models, reported for mouse and rats are desirable because data interpretation of the intrinsic allergenicity of wheat proteins becomes easier [38,48]. There are a number of adjuvant-free mouse models reported in the literature for tree nut, milk, sesame, and shellfish allergies [39,40,41,42,43,49,50]. Therefore, there is an exciting opportunity to develop novel adjuvant-free models for various types of wheat allergies.

Adjuvant-based models are also useful in studying mechanisms of wheat allergy because they will explain the immune responses to wheat proteins when the host is co-exposed to adjuvant-like factors present in the environment or food, such as enterotoxins [51,52,53]. However, in these models, the effects of adjuvant must always be differentiated from those induced by actual wheat proteins, making the experimental design and interpretations complicated [49,54,55,56]. Thus, adjuvant-based models are generally not preferred to evaluate the intrinsic allergenicity of food proteins per se [54,55,57]. However, they are used primarily to address the mechanisms of disease and to develop novel preventive/therapeutic agents.

##### Wheat Proteins to Use in Animal Testing: Which Ones?

As discussed earlier, wheat contains four groups of protein allergens that differ in solubility: Albumins, globulins, gliadins, and glutenins (Figure 2). A large number of wheat proteins (more than 40) show an allergenicity property in humans [18,44]. These allergenic proteins were identified in all four general categories of wheat proteins. A few examples of major wheat allergens in these four groups include the following: α-amylase inhibitor, lipid transfer protein (LTP), and globulin 3 are major water/saline-soluble wheat allergens; ω5-gliadin is a major alcohol-soluble wheat allergen; and high molecular weight glutenin is a major acid-soluble wheat allergen [18,44].

As noted earlier, both the rat and most mouse models, except Jin et al. (2017), who used salt-soluble wheat protein extract) used gliadins either as a gluten extract or purified gliadin protein [45]. However, human exposure to wheat via food or in occupational settings involves natural exposure to all four groups of wheat proteins at the same time. It is noteworthy that when using whole-wheat flour exposure in dogs, Buchanan and Frick (2002) reported an allergic response to all four wheat protein fractions [58]. Thus, it seems that it is possible to develop models with wheat flour exposure. Therefore, future small animal (mouse, rat) studies might consider simulating human exposure to wheat flour as a model improvement.

One challenge we faced in our interpretation of the literature across studies is the lack of information on the genotype of the wheat used in animal testing in many studies (Table 2). This is an important consideration because there are five different wheat genotypes: AA, BB (extinct now; SS is the closet relative), DD, AABB, and AABBDD [59]. Although the genotype is not identified in the models, our analysis identified that many models may have actually used the AABB genotype (Table 2). Theoretically, it is possible that different genotypes may differ in allergenicity. In fact, there is some evidence that different wheat genotypes may differ in their allergenicity and immune-toxicity potential [59]. Kohno et al. (2016) reported the identification of a hypoallergenic wheat line deficient in ω-5 gliadin, which is a major anaphylaxis causing wheat allergen [60]. Larre et al. (2011) evaluated the allergen content of diploid vs. hexaploid wheat genotypes and reported a lower allergen content and IgE reactivity of diploid wheat compared to hexaploid wheat [61]. Thus, different wheat genotypes may differ in their allergenic potential. Animal models are powerful tools to address this issue in the future.

Readouts of Sensitization and Disease

The gold standard readout of sensitization is wheat specific IgE antibody; measuring IgG1 alone is not useful (Table 2, Table 3, Table 4 and Table 5). However, IgE production suggestive of sensitization may or may not lead to actual disease. Therefore, it is critical to demonstrate disease readouts (such as anaphylaxis). It is also important to confirm that the disease is IgE-mediated in mice by measuring murine mucosal mast cell protease-1 levels in the blood [45,46]. 

### 2.3. Opportunities for Improvement of Current Models and Development of New Models

In our opinion, an ’ideal’ wheat allergenicity animal model would include, at the minimum, all the following features: (i) Robust readouts of wheat specific IgE antibody responses (i.e., sensitization); (ii) robust quantifiable readouts of wheat allergy disease elicitation (e.g., anaphylaxis and/or gut/skin/airways disease); and (iii) use of physiological routes (skin, eyes, airways, oral) of exposure to wheat proteins to induce sensitization as well as disease elicitation. Current models can be improved and new models can be developed taking into consideration these three fundamental characteristics of an ‘ideal’ animal model.

Currently, there are no animal models for occupational IgE-mediated wheat allergies, including allergic rhinitis, allergic conjunctivitis, baker’s asthma, and contact urticaria. There are also no wheat allergy disease elicitation rat models involving anaphylaxis, gut/skin/airways reactions. Rhesus monkeys have been used to conduct basic and applied studies on celiac disease, but not wheat allergies so far [62,63,64]. These represent opportunities for novel animal model development in wheat allergy.

### 2.4. What More Can Animal Models of Wheat Allergies Teach us? Anticipated Lessons

Animal models of wheat allergenicity can be used to address a number of questions, including the following as discussed. 

#### 2.4.1. Determination of Genetic Susceptibility Factors for Wheat Allergy

There are two contradicting reports showing wheat-dependent exercise-induced anaphylaxis (WDEIA) in a patient without filaggrin mutation and an association of mutation in the filaggrin gene with WDIEA in a Japanese family [65,66]. The available evidence showed that C3H/Hej and Balb/c mice strains have different susceptibilities to gliadin-induced allergy, suggesting genetic control of the disease [30]. There are extensive genetics data on celiac disease, where the role of MHC vs. non-MHC genetics has been elucidated [67,68,69]. Thus, animal models can also be employed to dissect the genetic basis of different types of wheat allergies.

#### 2.4.2. Identification of Environmental Factors in Wheat Allergies

Although genetic susceptibility is required to develop allergies in general, the relatively recent upsurge in allergies suggests the existence of environmental triggers of disease, which are likely be multifactorial in nature. A number of candidates are being considered, including the following: Role of detergents, cosmetics, antibiotics, anti-microbial compounds used in personal hygiene products (e.g., triclosan), food preservatives (e.g., tert butylhydroquinone), vitamin D, and the role of beneficial vs. harmful microbiomes [70,71,72,73,74,75,76]. Wheat allergenicity animal models will be vital to identify specific environmental factors that are required to develop or prevent wheat allergy (e.g., defining the timing and routes of exposure, dose effects, etc.).

#### 2.4.3. Determination of the Impact of Food Processing Methods on Wheat Allergenicity

There is extensive evidence in the literature that food processing can alter the allergenicity of food proteins [77,78,79]. However, a growing consensus is that thermal processing does not reduce or eliminate allergenicity of wheat allergens [77,79]. Nevertheless, there is promising data that suggest that fermentation and enzyme treatment may be able to reduce the allergenicity of wheat to produce hypo/non-allergenic wheat products [60,80,81,82,83,84,85]. Animal models can serve as very useful pre-clinical testing tools for this application. 

#### 2.4.4. Allergenicity Testing of Genetically Modified Wheat

As discussed earlier, five distinct wheat genotypes are known to contribute to the genetic diversity of the wheat crop [59]. In addition, using these genotypes, wheat breeders have successfully developed thousands of wheat varieties and wheat lines [59,86]. Furthermore, currently, efforts are also underway to genetically modify and produce engineered wheat lines [60,87]. Validated animal models will help in testing the allergenicity of new wheat developed by conventional breeding/selection and genetic engineering [39,61,88].

#### 2.4.5. Pre-Clinical Testing of Novel Pharmaceuticals and Vaccines for the Prevention and Treatment of Wheat Allergies

Animal models of wheat allergies can also serve as pre-clinical testing tools to develop novel pharmaceuticals as well as vaccines for these disorders [89,90]. For example, novel phytochemicals or synthetic molecules can be tested in animal models to develop new anti-wheat allergy medicines. Similarly, vaccines and immunotherapy protocols can be developed using validated animal models as pre-clinical testing tools [89,90].

## 3. Conclusions

Wheat allergies are a significant public health problem and food safety issue at the global level. Fundamental mechanisms underlying this problem are incompletely understood at present. A number of valuable animal models have been developed for wheat food allergy and anaphylaxis, but not for other types of wheat-induced allergies. Currently, animal models are markedly underutilized to advance mechanistic knowledge on wheat allergies. There are ample opportunities for further improvement of current models as well as to develop new models. Our analysis shows that animal models can provide insight into the IgE epitope structure of wheat allergens, effects of detergents and other chemicals on wheat allergenicity, and the role of genetics, microbiome, and food processing in wheat allergenicity. Although animal models have inherent limitations, they can serve as useful pre-clinical testing tools to develop safer genetically modified wheat, hypoallergenic wheat products, pharmaceuticals, and vaccines. Thus, validated animal models will be instrumental to advance basic and applied wheat allergenicity research to enable the development of effective prevention and control strategies for wheat allergy—a growing public health and food safety challenge of global proportions.

## Figures and Tables

**Table 1:** The dog and rat models of wheat allergy: experimental approaches used to study.

Model/Developers	Wheat Protein Used	Sensitization (Route, Dose, Age, Gender, Adjuvant)	Elicitation of Reaction (Route, Dose and Age)	Immune Markers	Disease Phenotype
Dog model *Spaniel/basenji* inbred dog colony/Buchanan et al. (1997)	Wheat + cow’s milk + beef extract	SC injection (on days of age: days 1, 22, 29, 50, 57, 78, and 85) 1 ug each of the food allergens + 0.2 mL alum; SC injection distemper-hepatitis vaccine on days 21, 49, and 77; Booster at bimonthly intervals with 10 ug each of the food allergens; Bleedings at 3, 4 months	At 6 months: Feeding challenge with 200 g wheat flour gruel or cow’s milk	Specific IgE, Skin prick test	Vomiting and/or diarrhea (increased number of loose or watery stools for 2–4 days after the feeding challenge)
Rat models *Brown Norway* inbred rats (bred on gluten-free diet for three generations)/Kroghsbo et al. (2014) *Brown Norway* inbred rats/Bellegaard et al. (2019)	Gluten (Unmodified, acid hydrolyzed, Enzyme hydrolyzed) Native gluten vs. acid hydrolyzed gluten	IP sensitization: day 0: 200 ug adsorbed on Alhydrogel/rat in PBS; Days 14, 21, and 28: 20 ug in 0.9% NaCl; 0.2 mL volume/bleeding on day 35	None	Specific IgE, IgG Rat Basophilic Leukemia cell degranulation in vitro	None
		Oral sensitization: Female BN rats; Days 1 to 35: gavage with 0.2, 2, and 20 mg suspension in 0.5 mL PBS; Bleeding on days 0, 14, 28, and 42	None	Specific IgE, IgG Rat Basophilic Leukemia cell degranulation in vitro	None
		Skin sensitization: damage to skin then apply gluten without adjuvant 3 times per week for 3–5 weeks	None	Specific IgE, IgG antibodies	None

IP = intra-peritoneal injection; SC = subcutaneous injection.

**Table 2:** Wheat food allergy mouse models: experimental approaches.

Model/Developers	Wheat Protein Used	Sensitization (Route, Dose, Age, Gender, Adjuvant)	Elicitation of Reaction (Route, Dose and Age)	Immune Markers	Disease Phenotype
*B10.A* model by Kozai et al. (2006)	Water/saline- soluble protein extract, Alcohol-soluble protein extract, alkali-soluble protein	IP (day 0, 14, 28, and 42) 10 ug + 1 mg alum/mouse Female B10.A	20 mg/0.5 mL/mouse oral feeding plus acute or moderate exercise	Specific IgE	Time to exhaustion, mucosal lesions in the small intestine, wheat protein leakage into the liver
*Balb/cJ B10.A C3H/HeJ* model by Bodinier et al. (2009)	Gliadin (Hardi)	IP (day 0, 10, 20, and 30) 10 or 20 ug + 1 mg alum/mouse 3-week females Balb/CJ 4–5-week females B10.A, C3H/HeJ	Nasal administration (10 ug on day 40)	Specific IgE, IgG1; IL-4, IL-5, IL-10, GM-CSF, IL-12 in lungs; cell counts in lung fluids	Eosinophil influx to lungs upon challenge
*B10.A* model by Tanaka et al. (2011)	Gliadin, purified ω5-gliadin	Gliadin 100 ug/mouse first IP injection; 50 ug/mouse for next 5 injections at weekly interval + 1 mg alum/mouse Female B10.A 5 weeks age	Gliadin at 0.1 and 0.8 mg/mouse/0.5 mL acetic acid; ω5 gliadin at 0.1 mg/mouse/0.5 mL acetic acid Oral feeding plus acute exercise	Specific IgE	Anaphylaxis by hypothermia shock response, voluntary exercise performance, leakage of wheat proteins into the blood
*Balb/cJ* model by Denery-Papini, et al. (2011)	Gliadins extract (Hardi) LTP1	IP (day 0, 10, 20, and 30) 10 ug of gliadins or LTP1 + 1 mg alum/mouse 3-week females	As in Bodinier et al. (2009)	Specific IgE	None reported
*Balb/cJ* model by Gourbeyre et al. (2012)	Deamidated gliadins (acid hydrolysis) (Hardi)	IP (day 0, 10, 20, and 30) 10 ug + 1 mg alum/mouse, 6-week females	IP injection with 1 mg + 1 mg alum on day 38	Total IgE, specific IgG1, IgG2a	None reported
*Balb/c* model by Adachi et al. (2012)	Acid hydrolyzed gluten	Skin sensitization (days 1–3, 8–10, 15–17, and 22–24) 0.5 mg	IP injection with 1 mg on days 18 or 25	Specific IgE, IgG1, plasma histamine levels	Hypothermia shock response
*Balb/c* model by Abe et al. (2014)	Native gliadin and deamidated gliadin by carboxylated cation exchange resin	IP (day 0, day 14) 50 ug of native gluten with 1 mg alum/mouse 5-week males	Intra-gastric administration of deamidated gliadin, 10 mg on days 28, 30, 32, 34, 36, 38, and 40	Specific IgE; peritoneal mast cells, histamine (gut and plasma)	Intestinal permeability, mast cell degranulation
*Balb/cJ* model by Jin et al. (2017)	Saline-soluble wheat protein (duram)	IP (days 0, 10, 24, and 40), 10 ug + 1 mg alum, 6–8-week females	IP injection with 0.5 mg, 1 week after last sensitization and repeated	Specific IgE, IgG1, total IgE murine mast cell protease-1, correlation analysis among readouts, cytokines, chemokines, adhesion molecule in skin lesion	Hypothermia shock response, atopic dermatitis, skin mast cell degranulation, mucosal mast cell mediator release

IP = intraperitoneal injection; LTP = lipid transfer protein.

**Table 3:** Pathogenic IgE binding peptide epitopes present in wheat identified using a mouse model.

Protein	Pathogenic IgE Binding Peptide Epitopes
*Salt-soluble protein*	
LTP1 *	(1) QARSQSDRQS; (2) GIARGIHNLN
*Alcohol-soluble proteins*	
α-gliadin	(1) PLVQQQ; (2) QQQFPGQQQQ ^#^; (3) YLQLQLP ^#^; (4) YPQQQPQYLQ; (5) SFQQPQQQYP
ω2-gliadin	(1) FPTPQQQFPE; (2) QQSFPLQPQQ ^#^; (3) QQLFPELQ
ω5-gliadin	(1) QQFPQQQ ^#^; (2) QQLPQQQ ^#^; (3) QQSPQQQ ^#^; (4) QQEFPQQQ; (5) QQQFPQQEFP

* LTP1 = Lipid transfer protein 1. #: Epitope is present in both the human and mouse model; Amino acids: Q = Glutamine; I = Isoleucine; P = Proline; F = Phenylalanine; G = Glycine; S = Serine; Y = Tyrosine; L = Leucine; E = Glutamic Acid; V = Valine; R =Arginine; N = Asparagine. Reference: [29].

**Table 4:** Major lessons learnt from animal models on wheat protein allergenicity.

Species	Wheat Allergen		Exposure Route	Sensitization	Elicitation of Reaction
*Dog*	Gliadins		IP	IgE	ND
	Glutenins Albumins Globulins	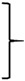	Oral	ND	Vomiting, Diarrhea
			Skin	ND	Skin Reaction
	Thioredoxin Modified Gliadins & Glutenins		Skin	ND	Reduced Skin Reaction
*Mouse*	Gliadins		IP	IgE	Anaphylaxis, EIA
	Gluten		Skin	No IgE	No Anaphylaxis
	Gluten + Detergent		Skin	IgE	Anaphylaxis
	Acid-Hydrolyzed gluten (AHG)		Skin	IgE	Anaphylaxis
	AHG + Detergent		Skin	Increased IgE	Increased Anaphylaxis
	Deamidated-Gliadins (DG)		IP	Increased IgE	ND
	DG		Oral	ND	Reduced Gut Reactions
	Albumins + Globulins		IP	IgE	Anaphylaxis
					Dermatitis (Th1, Th2, Th17 Cytokines + Allergenic Chemokines)
*Rat*	Gluten and AHG		IP	IgE	ND
			Skin	IgE	ND
	Enzyme Hydrolyzed Gluten		Oral	IgE	ND
			IP	IgE	ND
			Oral	IgE	ND

Abbreviations: IP = intraperitoneal injection; ND = not done; EIA = exercise-induced anaphylaxis.

**Table 5:** Animal models of wheat allergy: relative strengths and limitations.

Model Characteristics	Dog	Rat	Mouse
*Strengths*			
Protocol used in the model development is relatively simple	No	Yes	Yes
Used wheat flour for exposure	Yes	No	No
Used purified wheat allergens/extracts for exposure	No	Yes	Yes
Studied immune response to all four general groups of wheat allergens	Yes	No	No (most)
Used oral route to induce sensitization	No	Yes	No
Studied clinical reactions after oral exposure	Yes	No	No
*Limitations*			
Used adjuvant to induce sensitization	Yes	No	Yes
Used injection to induce sensitization	Yes	No	Yes
Used injection to elicit clinical reaction	Yes (skin)	No	Yes
Limited availability of animal breed/strain	Yes (Limited)	Commercially Available	Commercially Available
